# The Prevalence of Pediatric Septic Arthritis of the Hip with Concomitant Osteomyelitis: A Retrospective Study of 58 Consecutive Cases Investigated Using MRI

**DOI:** 10.2106/JBJS.25.01422

**Published:** 2026-05-05

**Authors:** Ahmer Ahmad Khan, Elio Paris, Viola Sbampato, Giacomo De Marco, Anne Tabard-Fougère, Oscar Vazquez, Christina Steiger, Romain Dayer, Dimitri Ceroni

**Affiliations:** 1 Division of Paediatric Orthopedics, Geneva University Hospitals and University of Geneva, Geneva, Switzerland; 2 Faculty of Medicine, University of Geneva, Geneva, Switzerland; 3 Department of Orthopaedics and Trauma Surgery, Geneva University Hospitals, Geneva, Switzerland

## Abstract

**Background:**

This observational study systematically used magnetic resonance imaging (MRI) to determine the prevalence of concomitant osteomyelitis and its influence on clinical outcomes in cases of pediatric septic arthritis (SA) of the hip.

**Methods:**

We retrospectively analyzed the demographic, clinical, microbiological, and radiographic data of 58 children treated for SA of the hip who underwent systematic MRI between 2000 and 2025. Patients were categorized into 2 groups: isolated septic arthritis and septic arthritis with concomitant osteomyelitis. The clinical and laboratory parameters, causative pathogens, and treatments were compared between the groups.

**Results:**

Concomitant osteomyelitis was identified with MRI in 43% (25) of the 58 patients, while radiographs detected it in only 16%. Demographic, clinical, and inflammatory parameters were statistically similar between the groups. *Kingella kingae* was the most commonly identified pathogen (37.9%), and *Staphylococcus aureus* and *Streptococcus* spp. were more frequently associated with repeat surgery. No significant differences in complication rates, treatment duration, or outcomes were found between the groups.

**Conclusions:**

The systematic use of MRI revealed concomitant osteomyelitis in >40% of cases of pediatric SA. However, the presence of osteomyelitis was not associated with worse outcomes, suggesting that factors related to the microorganism profile or virulence must contribute substantially to disease severity. Nonetheless, MRI should be considered early in any diagnostic work-up of pediatric SA of the hip.

**Level of Evidence:**

Diagnostic Level III. See Instructions for Authors for a complete description of levels of evidence.

In children, septic arthritis (SA) of the hip can occur in isolation or with concomitant osteomyelitis. The reported prevalence of osteomyelitis in pediatric SA of the hip ranges widely, from 12% to 75%^1-3^, but none of those studies had incorporated systematic magnetic resonance imaging (MRI) to evaluate this association. A recent systematic review reported osteomyelitis in 33.2% of cases of pediatric SA of the hip^4^. Subgroup analyses revealed prevalences of 36.6% in the U.S. versus 29% in Asia, and prevalences of 29.4% from 1976 to 2010 versus 35.2% from 2011 to 2023^4^. These differences were attributed to advances in medical imaging, particularly the greater availability of MRI^4^.

Moreover, it is accepted that children presenting with SA with concomitant osteomyelitis have less favorable outcomes and require both more surgical procedures and longer antibiotic therapy than patients with isolated SA^5-9^. Agarwal and Kumar^10^ even reported that concomitant proximal femoral osteomyelitis in children with SA of the hip was associated with more severe hip damage.

Understanding the prevalence of osteomyelitis in pediatric patients presenting with SA of the hip appears essential, as this information will influence surgeons’ decisions regarding surgical procedures and is essential to informing parents about potential adverse outcomes. The present study aimed to assess the true incidence of osteomyelitis in pediatric SA of the hip by systematically analyzing MRI data, in order to clarify the clinical and therapeutic implications of the osseous involvement.

## Materials and Methods

After approval by the ethics committee (2023-00578), we retrospectively reviewed the medical charts of every child and adolescent admitted to our institution with SA of the hip between January 2000 and June 2025. Eligible patients had confirmed SA of the hip, had received antibiotic treatment in our pediatric department, and had undergone an MRI evaluation.

Data on age, sex, and the bone segment involved in cases with concomitant osteomyelitis were collected for every patient. We also collected temperature at admission and laboratory values such as the white blood-cell (WBC) count, platelet count, erythrocyte sedimentation rate (ESR), and serum C-reactive protein (CRP) level. We assessed how well the Kocher criteria (temperature of ≥38.5°C, WBC count of >12,000/mm^3^, ESR of >40 mm/hr, refusal to bear weight or limping) and the Caird criteria (Kocher criteria + CRP of >20 mg/L) predicted isolated SA versus SA with osteomyelitis by estimating the sensitivity of the different items in these 2 algorithms^11,12^.

Blood samples were obtained from all patients and were systematically cultured, and bone aspirates and/or joint fluid samples were obtained and inoculated immediately before antibiotic administration. During 2000 to 2006, bacteria were identified solely from blood cultures and cultures of joint fluid or bone aspirate. From 2007 onward, both broad-range polymerase chain reaction (PCR) and *Kingella kingae*-specific real-time PCR were also routinely performed on joint fluid or bone aspirate samples for bacterial identification. In addition, since 2009, PCR of oropharyngeal swab samples has been used to detect *K. kingae* rtx genes in the oropharynx of infants at our institution^13^.

Every pediatric patient admitted to our department with suspected SA of the hip underwent an imaging work-up involving radiographs of both hips. All included patients also underwent MRI; images were acquired at 1.5 T (Avanto and Sola; Siemens), typically using the following 5 sequences: 3-dimensional STIR (short tau inversion recovery), T1-weighted turbo spin-echo in the longitudinal plane, T2-weighted plus fat suppression in 2 orthogonal planes, and a water-only data set of fast spin-echo T2-weighted Dixon sequences in the axial plane, as well as T1-weighted spin echo with frequency-selective fat saturation in 2 orthogonal planes after contrast injection (Dotarem; 0.2 mL/kg). MRI was performed according to a previously established protocol, but radiologists made the final choices regarding the most appropriate sequences and contrast enhancement agent to use. MRI in younger children was performed under sedation, and when surgery was indicated, patients were transferred directly to the operating room while still asleep. The radiographic and MRI data were retrospectively reviewed by a board-certified pediatric radiologist (15 years of experience), a senior pediatric orthopaedic surgeon (D.C., 30 years of experience), and a junior orthopaedist (A.A.K., 3 years of experience) until they reached a consensus diagnosis. MRI scans were analyzed to determine whether concomitant osteomyelitis was present and to identify its location, and the cases in which conventional radiography missed the osteomyelitis were counted.

All statistical analyses were performed using R software (version 4.4.1; R Foundation for Statistical Computing) and the RStudio interface (version 2024.09.0; Posit). The level of significance was set at p < 0.05, and 95% confidence intervals (CIs) and effect sizes were calculated.

The normality of continuous variables was tested using quantile-quantile (Q-Q) plots and the Shapiro-Wilk test. Patients were categorized into 2 diagnostic subgroups: those with isolated SA and those with SA plus osteomyelitis. Continuous outcomes were compared between groups using unpaired Mann-Whitney U tests, and non-normal distributions are reported as the median and interquartile range (IQR). Categorical outcomes were compared using Fisher exact tests and are reported as the number and percentage.

## Results

Twenty-two of the 80 patients initially identified were excluded due to not meeting inclusion criteria (primarily lack of MRI evaluation or incomplete data). Of the remaining 58 patients, 33 (57%) had isolated SA and 25 (43%) had SA plus osteomyelitis. The precise osteomyelitis locations are detailed in Figure 1-A. No significant demographic differences were observed between the groups without and with osteomyelitis (Table 1). Median ages were similar (27 versus 20 months, respectively; p = 0.577), as were the sex distribution (9 [27%] of 33 versus 8 [32%] of 25 female, p = 0.920) and laterality (18 [55%] of 33 versus 11 [44%] of 25 right-sided, p = 0.596).

**Fig. 1:**
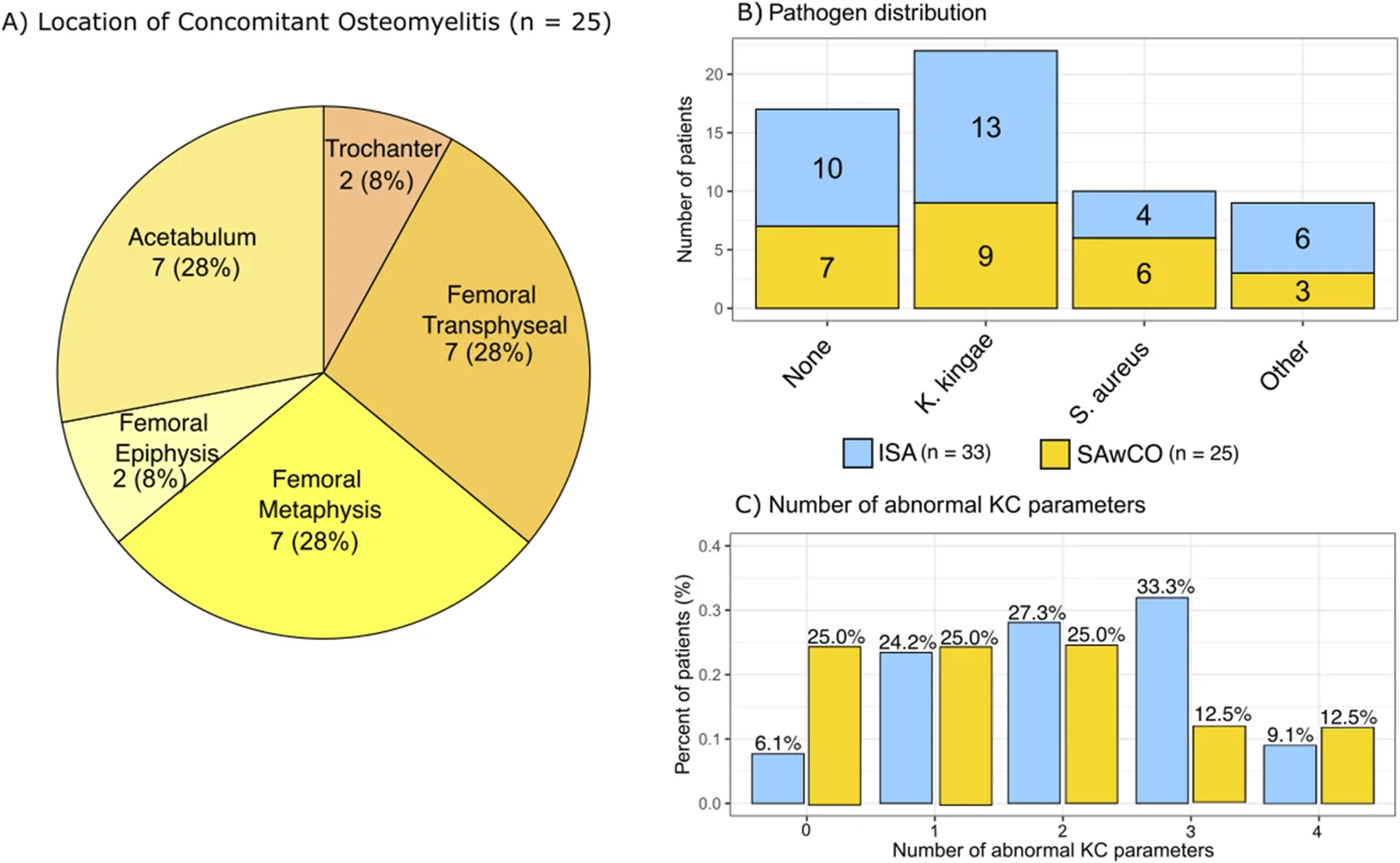
Characteristics of patients with isolated septic arthritis of the hip (ISA, n = 33) and septic hip arthritis with concomitant osteomyelitis (SAwCO, n = 25). **Fig. 1-A:** Anatomical location of osteomyelitis. **Fig. 1-B:** Distribution of the identified pathogens. **Fig. 1-C:** Number of abnormal Kocher and Caird (KC) criteria (temperature at admission of ≥38.5°C, WBC count of >12,000/mm^3^, ESR of >40 mm/hr, refusal to bear weight or limping, CRP of >20 mg/L).

**Table 1:** Demographic Characteristics of Children with Septic Hip Arthritis with and without Concomitant Osteomyelitis*

	Osteomyelitis		P Value	95% CI of the Difference†	Effect Size
	Yes (N = 25)	No (N = 33)			
Age *(mo)*	20 [11, 120]	27 [13, 53]	0.577	−10 to 33	0.026
Female	8 (32%)	9 (27%)	0.920	−23% to 32%	0.001
Right side	11 (44%)	18 (55%)	0.596	−19% to 40%	0.037
Age distribution					
0-12 months	7 (28%)	8 (24%)	0.983	−27% to 30%	<0.001
13-24 months	6 (24%)	8 (24%)	>0.999	−23% to 22%	<0.001
25-36 months	0 (0%)	3 (9%)	0.342	−22% to 4%	0.118
37-48 months	2 (8%)	5 (15%)	0.674	−27% to 13%	0.023
>48 months	10 (40%)	9 (27%)	0.459	−15% to 41%	0.072

* Values are given as the median [interquartile range] or as the number (percentage). P values were calculated using the Mann-Whitney U test for age and Fisher exact test for categorical variables.

† No-osteomyelitis group minus osteomyelitis group.

**Table 2:** Clinical and Biological Characteristics of Children with Septic Hip Arthritis with and without Concomitant Osteomyelitis*

	Osteomyelitis		P Value	Effect Size	95% CI of the Difference
	Yes (N = 25)	No (N = 33)			
Temperature *(°C)*	38.0 [36.8, 38.8]	37.9 [36.9, 38.5]	0.837	0.129	−0.5 to 0.7
WBC count *(×1,000/mm*^*3*^*)*	11.1 [8.3, 12.4]	12.5 [9.4, 15.6]	0.136	0.144	−4.2 to 0.5
CRP level *(mg/L)*	33.0 [14.0, 74.0]	35.7 [16.2, 83.5]	0.994	0.327	−22.0 to 20.0
ESR *(mm/hr)*	50.0 [28.5, 62.5]	34.5 [16.5, 52.5]	0.288	0.073	−7.0 to 23.0
Platelet count *(×1,000/mm*^*3*^*)*	411 [248, 472]	332 [257, 464]	0.391	0.036	−42 to 120

* Values as given as the median [interquartile range]. P values were calculated using using the Mann-Whitney U test. Temperature and CRP data were available for 25 with and 31 without osteomyelitis. ESR data were available for 18 with and 26 without osteomyelitis. Platelet count data were available for 24 with and 31 without osteomyelitis.

*K. kingae* was the most frequently identified pathogen, accounting for a total of 22 cases (13 of the 33 without and 9 of the 25 with osteomyelitis) (Fig. 1-B). In addition to *K. kingae* being the leading cause of SA in these young children, it is noteworthy that a high proportion of these cases (9 of 22, 41%) had associated osteomyelitis. Infections with *Staphylococcus aureus* also had a high rate of osteomyelitis (6 of 10, 60%). *S*. *aureus* and *Streptococcus* spp. infections were more frequently associated with the need for repeat surgical procedures.

Other pathogens (*Streptococcus* spp. and less common pathogens) also showed a non-negligible rate of osseous involvement (3 of 9, 33%). Before the advent of nucleic acid amplification assays and their use at our institution (2000 to 2006), only 9 cases of SA were recognized, and more than half of them were culture-negative infections.

The clinical and biological characteristics of the 2 groups at admission were statistically similar (p > 0.05), and the median temperature at admission and systemic inflammatory markers also demonstrated no clinically meaningful differences (Fig. 2).

**Fig. 2:**
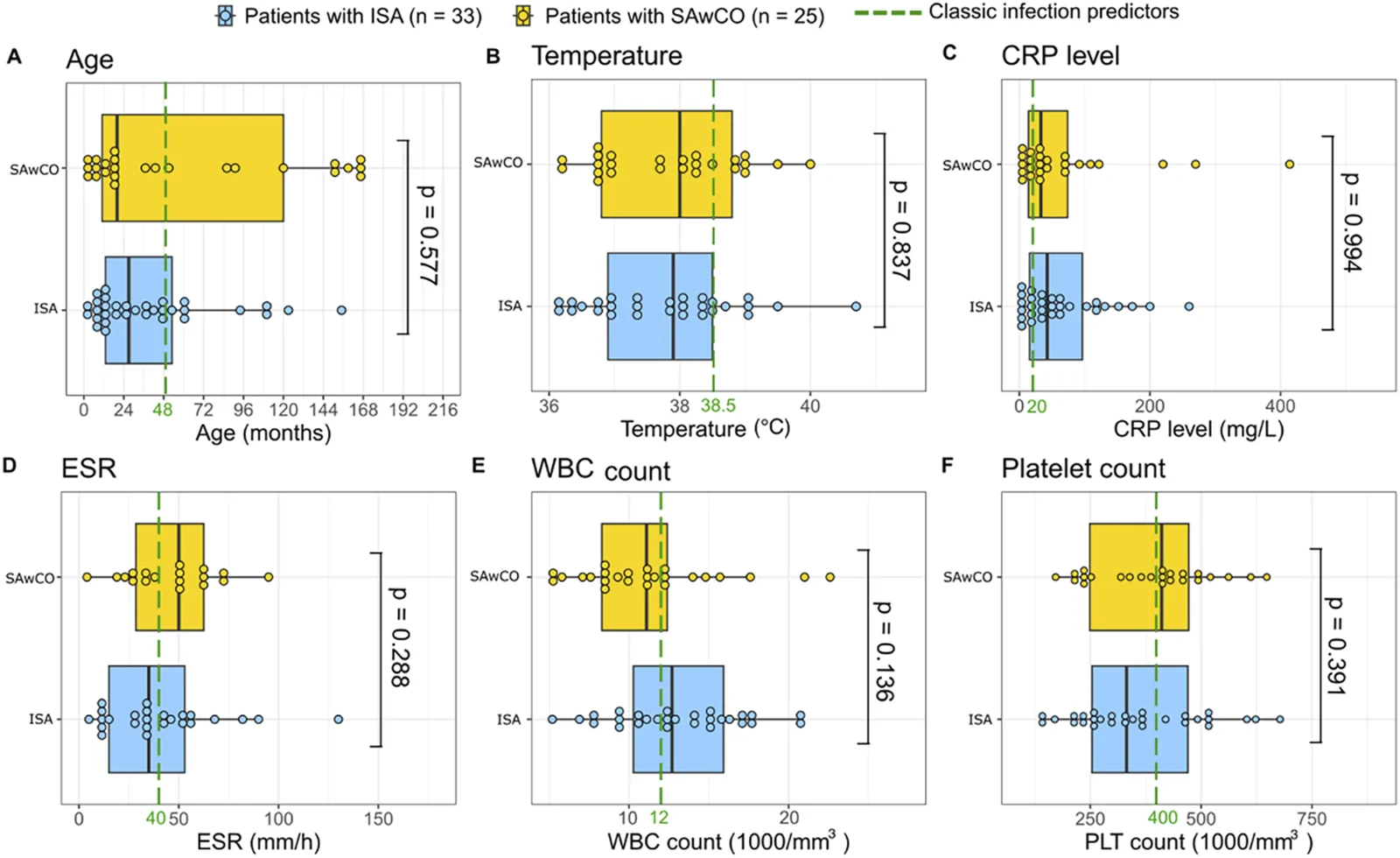
Comparison of clinical and biological infection markers between patients with isolated septic arthritis of the hip (ISA, n = 33) and septic arthritis with concomitant osteomyelitis (SAwCO, n = 25). The IQR is indicated by a box, the median is indicated by a line within the box, and the whiskers indicate points within 1.5 IQR widths of the box.The groups were compared using unpaired Mann-Whitney U tests. The vertical green lines designate the classic infection thresholds defined by Kocher and Caird. PLT = platelet.

The diagnostic utility of the classic infection criteria described by Kocher and of those modified by Caird was found to be limited in the present cohort (Table 3). The median number of predictors that were present was lower in patients with osteomyelitis (median, 1.63 [IQR, 0.75 to 2.25]) than in those with isolated SA (2.15 [1.00 to 3.00]), but not significantly so (p > 0.05). Furthermore, applying the Kocher and Caird criteria revealed a critical flaw in their predictive value (Fig. 1-C).

**Table 3:** Classification of Children Using the Kocher Criteria Versus Kocher and Caird Criteria

	Kocher: 4 Criteria*	Kocher and Caird: 5 Criteria†
No. of patients with data for all criteria	41	41
No. of applicable criteria present		
Mean	1.6	2.0
Median	2	2
Range	0-3	0-4
Distribution		
0	6 (14.6%)	4 (9.8%)
1	13 (31.7%)	10 (24.4%)
2	14 (34.1%)	11 (26.8%)
3	8 (19.5%)	12 (29.3%)
4	0 (0%)	4 (9.8%)
5	−	0 (0%)

* Kocher criteria: body temperature of ≥38.5°C, inability to bear weight or limping, WBC count of >12,000/mm^3^, ESR of >40 mm/hr.

† Kocher and Caird criteria: Kocher criteria plus CRP of >20 mg/L.

## Discussion

SA of the hip is a recognized pediatric condition that can lead to considerable morbidity and a risk of orthopaedic sequelae. For example, it is widely held that children with SA accompanied by osteomyelitis will have less favorable outcomes and require more surgical procedures and longer antibiotic therapy than patients with isolated SA or acute osteomyelitis^4,14-18^. These assumptions, however, have been based largely on expert opinion rather than on systematic investigations using MRI. The present work addresses this gap by presenting the largest pediatric case series of SA of the hip in which identification of concomitant infectious lesions of the bone was performed.

First, the present results provided robust evidence of the high prevalence of concomitant osteomyelitis in pediatric patients with SA of the hip that is revealed when this parameter is systematically evaluated using MRI. In the present series, 43% of patients with SA had accompanying osteomyelitis, a prevalence higher than that observed with conventional radiographs alone (16%). The reported rates of osteomyelitis accompanying SA varied substantially (from 12% to 75%) across previous studies^1-3^ due to differences in diagnostic methodologies and study designs. This variability highlights the essential role of MRI in identifying osseous involvement in anatomically complex regions such as the hip. Our observed prevalence aligned closely with the estimate of 33.2% from a recent meta-analysis^4^. Thus, the present findings support the early use of MRI for children with suspected SA of the hip, as it improves detection of concomitant osteomyelitis that can be missed on radiographs, enabling adjustments to monitoring and follow-up.

It is nevertheless important to point out that MRI results will not necessarily change the physician’s immediate antibiotic treatment or operative strategies. In fact, evidence that osteomyelitis is also present should not, by itself, be interpreted as an indication for routine bone drilling or more aggressive surgical management. Surgical decisions regarding osseous procedures, including repeat debridement, should primarily be based on the clinical course of the disease and the profile of the suspected causative microorganism (notably *S*. *aureus* or streptococci), rather than on anatomical MRI findings alone.

Second, the present findings contradict the conclusions of previous research seeking early clinical predictors of concomitant osteomyelitis in patients with pediatric SA of the hip. In our series, patients with and without osteomyelitis could not be distinguished by age, temperature, inflammatory blood markers, or the prevalence of *S*. *aureus.* Previous studies have linked concomitant osteomyelitis with older age, prolonged duration of symptoms, longer length of stay, and *S*. *aureus* infection^15^. Danilov et al.^19^ identified several independent predictors, such as an inability to bear weight and/or the presence of hip pain in children who were not yet walking, CRP level of >2 mg/dL, age of ≥4 years, and symptom duration of >4 days. Finally, many authors have recommended that MRI be considered in cases of pediatric SA, particularly for patients presenting with pain for >4 days, a scenario in which 96% of children were diagnosed with concomitant osteomyelitis^20^. These findings from previous studies suggest that traditional clinical parameters are insufficient for detecting deeper or adjacent infectious foci, supporting the use of MRI as the first-line means of imaging when SA of the hip is suspected. As documented by Murphy et al.^21^ and Agarwal and Kumar^10^, the early identification of concomitant osteomyelitis is both diagnostically and prognostically relevant.

Third, our results did not support the hypothesis that SA plus osteomyelitis is associated with less favorable outcomes and that it more frequently requires surgical procedures and longer antibiotic therapy than isolated SA. Previous studies, such as by Montgomery et al.^15^, had indicated that children with concomitant osteomyelitis experienced significantly less favorable outcomes and required more operations and longer hospitalizations, reinforcing the widely held view that osteomyelitis accompanying SA worsens the prognosis. Our findings diverged from this hypothesis, as the outcomes of the isolated and concomitant cases in our cohort were very similar. This suggests that factors related to specific pathogens may be more important contributors to the course of the disease. The discrepancy highlights the need for multicenter, prospective studies that could account for the microbial etiology of infections when evaluating the prognosis.

Fourth, our results demonstrated that the relevant classic clinical prediction algorithm (the Kocher and Caird criteria) was of limited diagnostic utility for discriminating between SA with and without osteomyelitis. In our study, having fewer positive predictors did not exclude a more complex disease, and these algorithms lacked sufficient sensitivity and specificity to identify cases with concomitant osteomyelitis. These findings are consistent with recent literature showing that these criteria, developed before the widespread recognition of *K. kingae* as a frequent cause of pediatric osteoarticular infections, are less effective in contemporary cohorts^22-25^.

Our study has some limitations inherent to its retrospective design, which increased the potential for missing data and selection bias. Although medical coding errors could have led to missed cases, our institution’s well-established coding system makes this unlikely. A key limitation is that children with SA of the hip who did not undergo MRI were excluded from the study; this could have influenced the results of the study. Because symptom onset could not be reliably determined retrospectively, detection of osseous involvement by MRI may have been affected. Indeed, during the first period of the study, emergency access to MRI was more difficult to obtain, with the consequence that some children with SA were treated without MRI being performed, which may have skewed our results. A third limitation stems from the fact that significant major improvements in microbiological analysis have taken place over time. The study covered a period (2000 to 2025) during which new nucleic acid amplification assays were progressively introduced. As a result, *K. kingae* infections were likely underdetected in the early diagnostic era, and the observed pathogen distribution partially reflects the improved diagnostics of the later period. Knowing that osteomyelitis was very frequently encountered in cases of SA due to *K. kingae,* it is reasonable to suspect that the study has underestimated the incidence of osseous lesions.

Despite these limitations, the descriptive data examined provided detailed information on the types and locations of these infections, as well as on the pathogens responsible for them. The use of a standardized MRI protocol, systematic data collection, and a review of the images by a multidisciplinary team represent notable strengths. Further prospective, multicenter studies are needed to confirm these findings and to identify reliable early diagnostic indicators of concomitant osteomyelitis.

### Conclusions

The prevalence of concomitant osteomyelitis in cases of pediatric SA of the hip was higher than expected (>40%) when this parameter was systematically evaluated using MRI. Traditional inflammatory markers were unreliable predictors of osteomyelitis accompanying SA, and *K. kingae* emerged as the leading causative microorganism. Our findings challenge the prevailing view that osseous involvement worsens the prognosis, suggesting instead that factors related to the causative pathogen may be more important determinants of severity. However, in our opinion, MRI should be considered early in the diagnostic work-up for pediatric SA of the hip, primarily to stage the extent of the disease, detect adjacent foci, support differential diagnosis, and guide personalized monitoring and counseling. Future multicenter, prospective studies are needed to confirm these results and to establish pathogen-specific prognostic markers.
